# The marine sponge *Agelas citrina* as a source of the new pyrrole–imidazole alkaloids citrinamines A–D and *N*-methylagelongine

**DOI:** 10.3762/bjoc.11.220

**Published:** 2015-10-29

**Authors:** Christine Cychon, Ellen Lichte, Matthias Köck

**Affiliations:** 1 Alfred-Wegener-Institut, Helmholtz-Zentrum für Polar- und Meeresforschung, Am Handelshafen 12, 27570 Bremerhaven, Germany

**Keywords:** *Agelas citrina*, marine sponges, mauritiamine, NMR, pyrrole–imidazole alkaloids

## Abstract

The chemical investigation of the Caribbean sponge *Agelas citrina* revealed four new pyrrole–imidazole alkaloids (PIAs), the citrinamines A–D (**1**–**4**) and the bromopyrrole alkaloid *N*-methylagelongine (**5**). All citrinamines are dimers of hymenidin (**6**) which was also isolated from this sponge as the major metabolite. Citrinamines A (**1**) and B (**2**) are derivatives of the PIA dimer mauritiamine (**7**), whereas citrinamine C (**3**) is derived from the PIA dimer nagelamide B (**8**). Citrinamine D (**4**) shows an uncommon linkage between the imidazole rings of both monomeric units as it is only observed in the benzocyclobutane ring moiety of benzosceptrins A–C (**9**–**11**). Compound **5** is the *N*-methyl derivative of agelongine (**12**) which consist of a pyridinium ring and an ester linkage instead of the aminoimidazole moiety and the common amide bond in PIAs.

## Introduction

**Figure 1:**
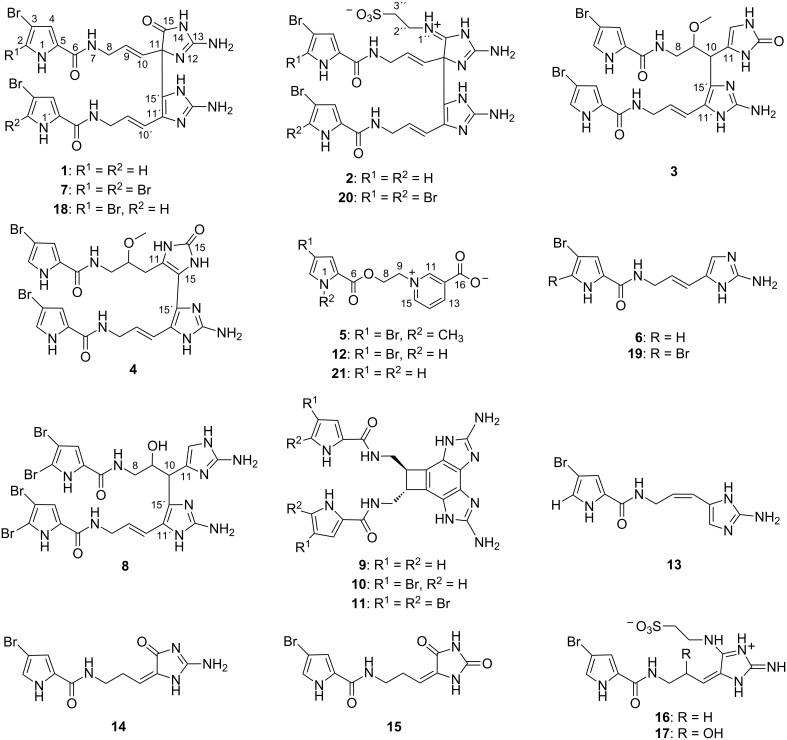
Selected pyrrole-imidazole alkaloids (**1**–**4**, **6**–**11**, and **13**–**20**), and agelongine analogues (**5**, **12**, and **21**).

The family of pyrrole-imidazole alkaloids (PIAs) represents a fascinating example of a large variety of secondary metabolites produced exclusively by marine sponges. To date, more than 150 PIAs have been isolated mainly from various species of the families *Agelasidae, Axinellidae, Dyctionellidae*, and *Hymeniacidonidae* and some of them show promising biological activities [1]. The chemical investigation of the Caribbean sponge *Agelas citrina* yielded the following known monomeric PIAs: hymenidin (**6**) [2], keramadine (**13**) [3], dispacamide B (**14**) [4], mukanadin B (**15**) [5], 2-debromotaurodispacamide A (**16**) [6], tauroacidin B (**17**) [7], and the dimeric PIA benzosceptrin B (**10**) [8–10] (Figure 1). Additionally, five new compounds, four with a dimeric PIA structure (**1**–**4**) and the *N*-methyl analogue (**5**) of the bromopyrrole alkaloid agelongine (**12**) [11], were isolated. Herein, we describe the isolation and structure elucidation of citrinamines A (**1**), B (**2**), and *N*-methylagelongine (**5**). The identification of citrinamines C (**3**) and D (**4**), which were both only obtained as mixtures, will also be discussed in this context.

## Results and Discussion

The crude extract of the sponge *Agelas citrina* (collected in the Bahamas in March 2001) was investigated by a standard separation scheme. The MeOH/CH_2_Cl_2_ extract of the sample was partitioned between *n*-hexane, *n*-BuOH, and H_2_O. The *n*-BuOH soluble fraction was further purified by size exclusion chromatography (Sephadex LH-20) and preparative reversed-phase HPLC yielding five new compounds (**1**–**5**). The isolation and structure elucidation of compounds **1** to **5** are discussed in detail.

**Figure 2:**
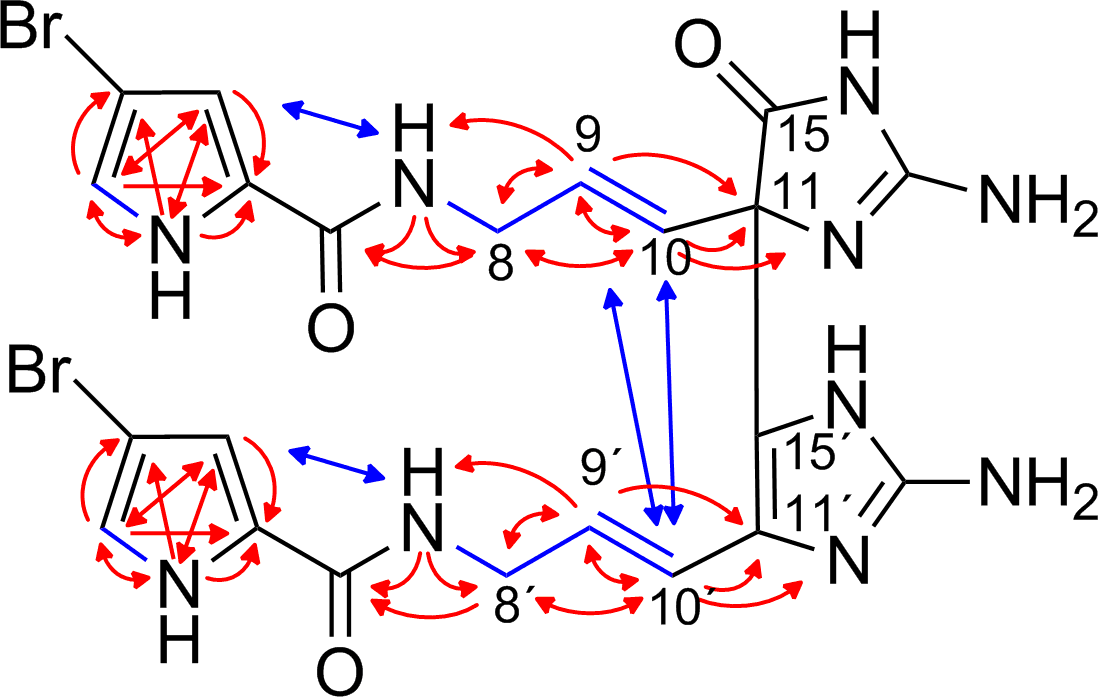
Selected ^1^H,^1^H-COSY (blue bonds), ^1^H,^1^H-NOESY (blue arrows), ^1^H,^13^C-HMBC, and ^1^H,^15^N-HMBC (both red arrows) correlations for citrinamine A (**1**).

The molecular weight of citrinamine A (**1**) was obtained from ESI mass spectrometry (HR–ESIMS, *m*/*z* 633.0330, [M + H]^+^, monoisotopic), together with the pseudomolecular ion peaks at *m*/*z* = 633/635/637 (1:2:1) the molecular formula C_22_H_23_Br_2_N_10_O_3_ was derived. The structure of **1** is very similar to mauritiamine (**7**) [12] and its 2´-debromo derivative nagelamide P (**18**) [13]. The 1D and 2D ^1^H and ^13^C NMR spectra of **1** showed two additional signals for sp^2^ methines indicating two 3-bromopyrrole carboxamide moieties (Table 1). The similarities of the NMR data of **1** with those of mauritiamine (**7**) and nagelamide P (**18**) proved the same connectivity of the respective monomers hymendin (**6**) and/or oroidin (**19**) (Figure 2). The linkage of both monomeric units between the sp^3^ quaternary C-11 (65.0 ppm) and the sp^2^ quaternary C-15´ (117.4 ppm) is indicated by the ^1^H,^1^H-NOESY [H-9 (5.87 ppm) to H-10´ (6.47 ppm) and H-10 (5.81 ppm) to H-10´] and the ^1^H,^13^C-HMBC correlations [H-9 to C-11, H-10 to C-11, H-9´ (6.10 ppm) to C-11´ (122.5 ppm), and H-10´ to C-11´] as well as by the absence of the H-15´ signal in the 1D ^1^H NMR spectrum. The missing signal for H-15 in the 1D ^1^H NMR spectrum and a sp^2^ quaternary carbon (δ_C_ 171.7 ppm) in the 1D ^13^C spectrum verified the oxidation of one aminoimidazole ring at position C-15 like in mauritiamine (**7**) and nagelamide P (**18**).

**Table 1:** ^1^H, ^13^C, and ^15^N chemical shifts of citrinamine A (**1**) (600 and 850 MHz, DMSO-*d*_6_) in comparison to mauritiamine (**7**) (600 MHz, DMSO-*d*_6_) and nagelamide P (**18**) (500 MHz, DMSO-*d*_6_).

Pos.	citrinamine A (**1**)^a^		mauritiamine (**7**)^a^		nagelamide P (**18**) [13]	

	δ_H_, mult. (*J*/Hz)	δ_C_/δ_N_	δ_H_, mult. (*J*/Hz)	δ_C_/δ_N_	δ_H_, mult. (*J*/Hz)	δ_C_/δ_N_

1-NH	11.80, s	(161)	12.66, s		12.68, brs	
2	7.02, dd (1.5; 2.8)	121.1	–	104.6	–	104.6
3	–	95.0	–	97.9	–	98.0
4	6.87^b^	111.3	6.95, brs	112.8	6.95, brs	112.8
5	–	127.0	–	127.9	–	128.4
6	–	159.5	–	158.9	–	159.5
7-NH	8.39, t (5.9)	(104)	8.39, t (6.1)		8.42, brt (5.9)	
8	3.90, dd (4.8; 9.6)	39.1	3.88, m	39.2	3.87, brt (5.3)	40.6
9	5.87, m	130.6	5.82^b^	128.9	6.02, dt, (16.0; 5.3)	139.1
10	5.81, d (15.8)	125.4	5.81^b^	126.4	6.05, brd	126.7
11	–	65.0	–	65.4	–	69.9
12-NH		(101)				
13	–	147.5	–	148.0	–	147.7
13-NH_2_					7.62, brs	
14-NH					12.12, brs	
15	–	171.7	–	175.0	–	183.4
1´-NH	11.80, s	(161)	12.66, s		11.80, brs	
2´	6.99, dd (1.4; 2.8)	121.1	–	104.6	6.98, brs	121.4
3´	–	95.0	–	97.9	–	95.0
4´	6.89^b^	111.3	6.85, brs	112.8	6.86, brs	111.7
5´	–	127.0	–	127.9	–	126.6
6´	–	159.4	–	158.7	–	158.8
7´-NH	8.41, t (5.8)	(107)	8.42, t (5.7)		8.39, brt (5.9)	
8´	4.01, m	40.1	3.97, m	40.1	3.95, m	40.5
9´	6.10, m	129.0	6.05, m	128.3	6.02, dt (16.0; 5.3)	136.6
10´	6.47, d (15.8)	116.8	6.48	116.4	6.41, brd (16.0)	115.7
11´	–	122.5	–	121.4	–	119.2
12´-NH		(132)			12.56, brs	
13´	–	147.5	–	148.0	–	147.7
13´-NH_2_					7.62, brs	
14´-NH						
15´	–	117.4	–	117.5	–	121.3

^a1^H and ^13^C chemical shifts are referenced to the DMSO-*d*_6_ signal (2.50 ppm and 39.5 ppm, respectively). ^15^N NMR shifts were not calibrated with an external standard. Therefore, the δ value has an accuracy of about 1 ppm in reference to NH_3_ (0 ppm) and the ^15^N NMR shifts are given without decimals. ^b^No multiplicity information could be given because of overlapped signals.

Based on ^1^H,^1^H-NOESY correlations H-8 (3.90 ppm) to H-10 and H-8´ (4.01 ppm) to H-10´ and the corresponding coupling constants for H-9/H-10 and H-9´/H-10´ (both 15.8 Hz), both of the two double bonds C-9 (130.6 ppm) and C-10 (125.4 ppm) as well as C-9’ (129.0 ppm) and C-10’ (116.8 ppm) were assigned to the *E* configuration. The same double bond geometries were also observed for mauritiamine (**7**) and nagelamide P (**18**). Citrinamine A (**1**) is the 2,2´-didebromo derivative of mauritiamine (**7**) and as in the original publications of **7** and **18** no chiroptical effect was observed for **1**. Synthetic studies on mauritiamine (**7**) [14] demonstrated the formation of similar racemic products by a chemical oxidative dimerization which could be an alternative origin of these metabolites.

**Figure 3:**
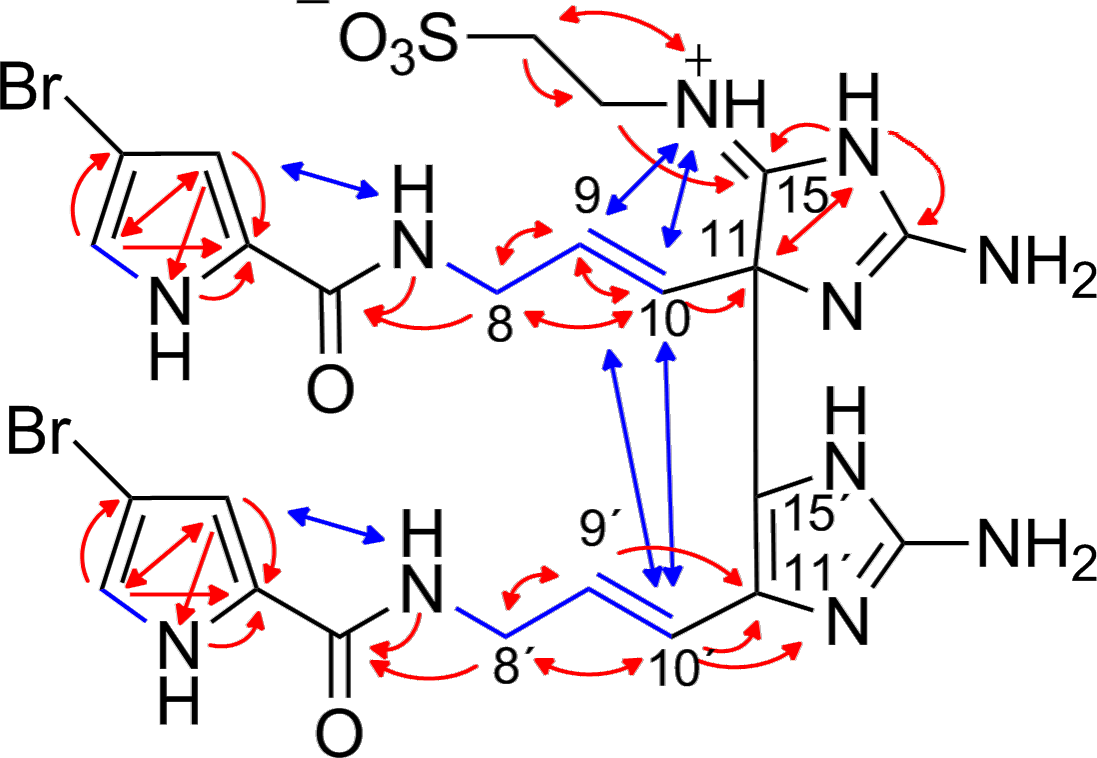
Selected ^1^H,^1^H-COSY (blue bonds), ^1^H,^1^H-NOESY (blue arrows), ^1^H,^13^C-HMBC, and ^1^H,^15^N-HMBC (both red arrows) correlations for citrinamine B (**2**).

The molecular formula of citrinamine B (**2**) was established by HR–ESIMS (*m*/*z* 740.0337, [M + H]^+^, monoisotopic) and the pseudomolecular ion peaks at *m*/*z* = 740/742/744 (1:2:1) to be C_24_H_28_Br_2_N_11_O_5_S. The 1D ^1^H and ^13^C NMR spectra of **2** were similar to those observed for **1**, except for the additional signals of one amine and two methylene groups (Table 2). The analysis of the ^1^H,^1^H-COSY and the ^1^H,^13^C-HMBC spectra linked these new signals to an aminoethyl chain and the comparison with the molecular formula indicated the existence of a taurine moiety in compound **2**. This structural proposal for citrinamine B (**2**) was proven by the ^1^H,^13^C-HMBC correlations H-2´´ (3.69/3.55 ppm) to C-3´´ (48.6 ppm) and C-15 (178.0 ppm), the ^1^H,^15^N-HMBC correlations H-3´´ (2.89/2.80 ppm) to N-1´´ (116 ppm), and the ^1^H,^1^H-NOESY correlation H-9/10 (5.95 ppm) to H-1´´ (9.85 ppm) (Figure 3).

**Table 2:** ^1^H, ^13^C, and ^15^N chemical shifts of citrinamine B (**2**) (600 and 850 MHz, DMSO-*d*_6_) in comparison to nagelamide H (**20**) (600 MHz, DMSO-*d*_6_).

Pos.	citrinamine B (**2**)^a^		nagelamide H (**20**) [15]	

	δ_H_, mult. (*J*/Hz)	δ_C_/δ_N_	δ_H_, mult. (*J*/Hz)	δ_C_/δ_N_

1-NH	11.81, s	(161)	11.71, s	
2	7.05, dd (1.4; 2.8)	121.7	–	104.8
3	–	95.4	–	97.9
4	6.90, dd (1.7; 2.4)	111.8	7.00, s	113.0
5	–	126.8	–	127.8
6	–	160.0	–	158.8
7-NH	8.44, t (5.7)	(104)	8.12, t (5.9)	
8	3.92^b^	39.7	3.86, m^c^; 4.04, m^c^	40.1^c^
9	5.95^b^	130.6	6.02, dt (6.0; 15.2)^c^	124.7^c^
10	5.95^b^	124.4	6.15, d (15.2)^c^	115.4^c^
11	–	68.0	–	112.8^c^
12-NH				
13	–	167.7	–	167.3
13-NH_2_	9.10, s; 8.59, s	(90)	8.79, brs; 9.11, brs.	
14-NH	10.04, s	(109)	10.19, brs	
15	–	178.0	–	177.4
1´-NH	11.80, s	(161)	12.72, s	
2´	7.01, dd (1.4; 2.8)	121.7	–	104.8
3´	–	95.2	–	98.0
4´	6.93, dd (1.7; 2.3)	111.8	6.97, s	113.0
5´	–	126.8	–	127.9
6´	–	159.8	–	158.8
7´-NH	8.48, t (5.8)	(106)	8.21, t (5.9)	
8´	4.15, m; 3.91^b^	39.9	3.92, m^c^	39.3^c^
9´	6.14, m	130.7	5.99, dt (6.2; 15.3)^c^	130.8^c^
10´	6.26, d (16.1)	115.2	5.90, d (15.3)^c^	129.6^c^
11´	–	124.5	–	69.7^c^
12´-NH		(132)	12.60, brs	
13´	–	147.7	–	148.3
13´-NH_2_			7.74, brs	
14´-NH			13.02, brs	
15´	–	117.5	–	123.1
1´´-NH	9.85, s	(116)	9.89, brs	
2´´	3.69, m; 3.55, m	41.0	3.65, m; 3.65, m	40.3
3´´	2.89, m; 2.80, m	48.6	2.81, t (7.1)	48.2

^a1^H and ^13^C chemical shifts are referenced to the DMSO-*d*_6_ signal (2.50 ppm and 39.5 ppm respectively). ^15^N NMR shifts were not calibrated with an external standard. Therefore, the δ value has an accuracy of about 1 ppm in reference to NH_3_ (0 ppm) and the ^15^N NMR shifts are given without decimals. ^b^No multiplicity information could be given because of overlapped signals. ^c^Possible assignment error, see text for details.

The structure of **2** is very similar to nagelamide H (**20**) [15] which is a mauritiamine derivative with a taurine residue in position C-15. There is probably an assignment error of the carbons C-8 to C-11 and C-8´ to C-11´ in the original publication of nagelamide H (**20**). All mauritiamine derivatives have comparable chemical shifts of these moieties and therefore a mixing up is plausible.

Based on NOESY correlations H-8 (3.92 ppm) to H-9/10 and H-8´ (4.15/3.91 ppm) to H-10´ (6.26 ppm) as well as the corresponding ^3^*J*_HH_ coupling constant H-9´ (6.14 ppm)/H-10´ (16.1 Hz), both of the double bonds of **2** were assigned to *E* configuration. Citrinamine B (**2**) is the 2,2´-didebromo derivative of nagelamide H (**20**). We could not observe a chiroptical effect for **2** as it was also described in the original work of **20**.

**Figure 4:**
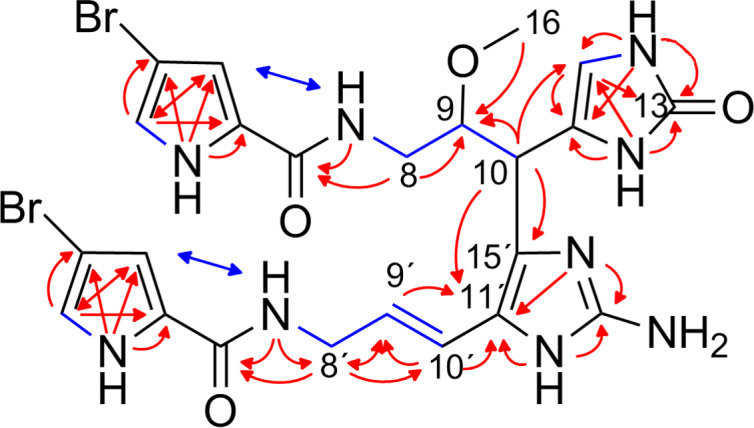
Selected ^1^H,^1^H-COSY (blue bonds), ^1^H,^1^H-NOESY (blue arrows), and ^1^H,^13^C-HMBC (red arrows) correlations for citrinamine C (**3**).

**Figure 5:**
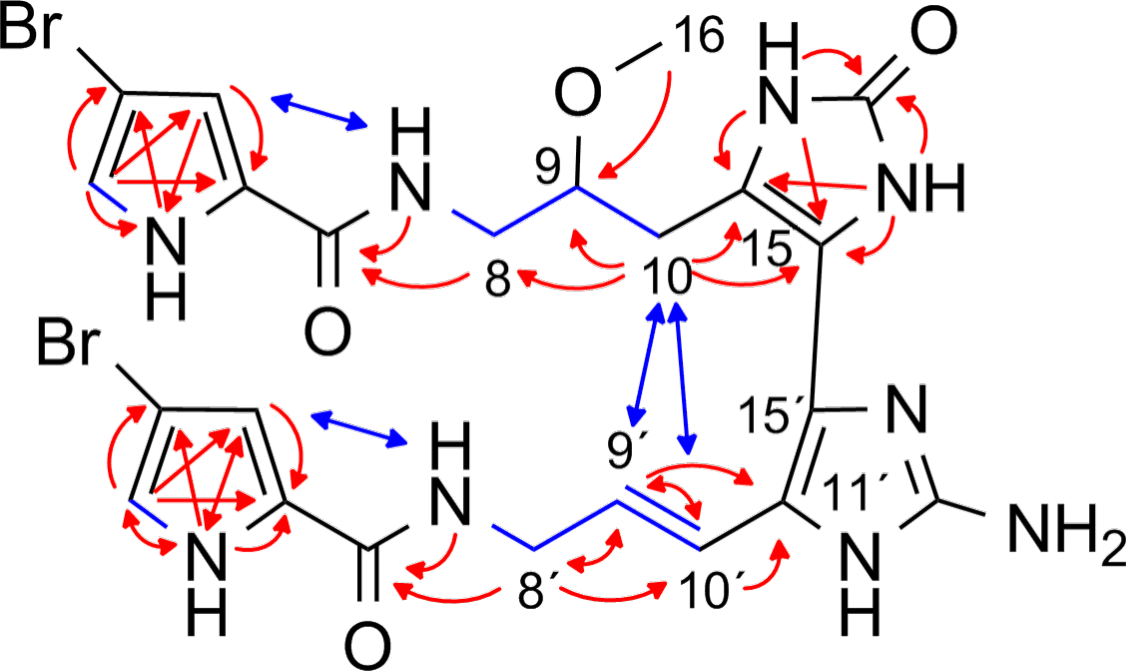
Selected ^1^H,^1^H-COSY (blue bonds), ^1^H,^1^H-NOESY (blue arrows), ^1^H,^13^C-HMBC, and ^1^H,^15^N-HMBC (both red arrows) correlations for citrinamine D (**4**).

Our investigation on *Agelas citrina* yielded two additional pyrrole–imidazole alkaloids, citrinamines C (**3**) and D (**4**), which were both obtained as mixtures. The isolation of the pure compounds of **3** and **4** by preparative chromatography failed but the analysis of the mixtures allowed the identification of their structures. The molecular weights of citrinamine C (**3**) (*m/z* 650.0493, [M + H]^+^, monoisotopic) and citrinamine D (**4**) (*m*/*z* 650.0470, [M + H]^+^, monoisotopic), and the respective pseudo-molecular ion peaks at *m/z* = 650/652/654 (1:2:1) indicated the molecular formula C_23_H_26_Br_2_N_9_O_4_ for both compounds. The analysis of the 1D and 2D ^1^H and ^13^C NMR spectra revealed for both compounds a dimeric hymenidin structure (Figure 4, Figure 5 and Supporting Information File 1, Table S1).

The ^1^H,^13^C-HMBC correlations of citrinamine C (**3**) from H-10 (4.09 ppm) to C-11´ (121.3 ppm) and C-15´ (117.9 ppm), suggested a connection of C-10 (34.5 ppm) with the imidazole ring of the second subunit as it was also described for nagelamide B (**8**) [15]. The structure of citrinamine C (**3**) was elucidated to be the 2,2´-didebromo derivative of nagelamide B (**8**) with an additional methylation of the hydroxy group at C-9 (80.8 ppm) and an oxidation of the imidazole ring at position C-13 (154.6 ppm) (an urea instead of a guanidine moiety). The relative configuration of the stereogenic centers C-9 and C-10 was identical as described for nagelamide B (**8**).

In contrast to citrinamine C (**3**), a different connection of the monomeric units was found for citrinamine D (**4**). Furthermore, **3** and **4** have an additional methoxy group at C-9 (80.8 and 78.1 ppm, respectively) and an oxidized imidazole ring compared to **1**. The ^1^H,^13^C-HSQC spectrum of **4** disclosed for C-10 (27.2 ppm) a methylene group (δ_H_ 2.34 ppm) and no hydrogen signal for C-15 (105.5 ppm). The missing proton signals for C-15 and C-15´ (115.8 ppm) and the ^1^H,^1^H-NOESY peaks H-10/H-9´ (6.16 ppm) and H-10/H-10´ (6.14 ppm) indicated a connection of the oxidized imidazole and the aminoimidazole ring in positions C-15 and C-15´. This uncommon linkage occurred so far only in the benzocyclobutane ring system of the benzosceptrins A–C (**9**–**11**). The incorporation of methoxy groups at carbon C-9 in citrinamines C (**3**) and D (**4**) may be attributed to the utilization of MeOH as solvent for the extraction of the sponge. To verify the structure elucidation of citrinamines C (**3**) and D (**4**), further extraction of sponge tissue of *Agelas citrina* is necessary to obtain pure material.

**Figure 6:**
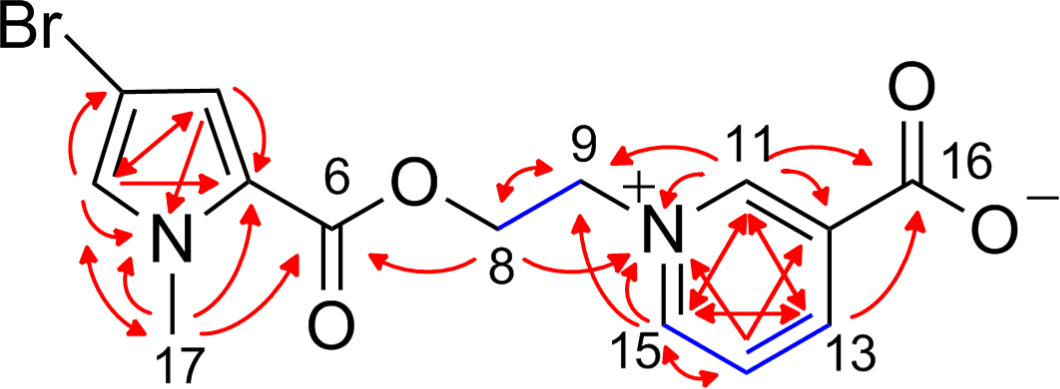
Selected ^1^H,^1^H-COSY (blue bonds), ^1^H,^13^C-HMBC, and ^1^H,^15^N-HMBC (both red arrows) correlations for *N*-methylagelongine (**5**).

The exact molecular weight of compound **5** was determined as *m*/*z* 353.0135 ([M + H]^+^, monoisotopic) corresponding to the molecular formula C_14_H_14_Br_2_N_2_O_4_. The pseudomolecular ion peaks at *m*/*z* = 353/355 (1:1) proved the presence of one bromine atom in **5**. The detailed analysis of the 1D ^1^H and ^13^C NMR spectra of **5** revealed only signals for the 3-bromopyrrole ring (two quaternary carbons at δ_C_ 121.6 ppm/94.1 ppm and two sp^2^ methines at δ_C_ 130.2 ppm/118.9 ppm) but no signals for the aminoimidazole moiety (Table 3). The ^1^H,^13^C-HMBC correlations from H-2 (7.30 ppm) to C-17 (36.5 ppm), H-17 (3.75 ppm) to C-2 (130.2 ppm), and H-17 to C-5 (121.6 ppm) indicated a methylation of the pyrrole nitrogen (Figure 6) which was proven by the ^1^H,^15^N-HMBC correlations H-2 to N-1 (159 ppm), H-4 (6.91 ppm) to N-1, and H-17 to N-1. The correlation between the methyl group H-17 and C-6 (158.6 ppm) suggested a linkage with the carboxylic acid in position C-5. The ^1^H,^1^H-COSY spectrum showed the connectivity of the methylene groups H-8 (4.65 ppm) and H-9 (5.07 ppm) and the ^1^H,^13^C-HMBC correlation H-8 to C-6 linked the ethylene group with the *N*-methyl-3-bromopyrrole carboxylic acid via an ester bond. The 1D ^1^H NMR spectrum of **5** disclosed four additional aromatic protons and ^1^H,^1^H-COSY peaks connected H-13 (8.95 ppm) with H-14 (8.25 ppm) and H-15 (9.26 ppm). The multiplet pattern of the four proton signals (*s* for H-11, *d* for H-14 and H-16, and *dd* for H-15) proved a *meta* di-substituted aromatic ring in the molecule. This is further proven by ^1^H,^13^C-HMBC correlations, such as H-11 (9.62 ppm) to C-12 (128.0 ppm), C-13 (145.7 ppm), and C-15 (146.6 ppm), H-13 and H-15 to C-11 (146.5 ppm), and H-14 to C-12.

**Table 3:** ^1^H, ^13^C, and ^15^N chemical shifts of *N*-methylagelongine (**5**) (600 MHz, DMSO-*d*_6_) in comparison to agelongine (**12**) (500 MHz, MeOH-*d*_4_) and daminin (**21**) (400 and 500 MHz, DMSO-*d*_6_).

Pos.	*N*-methylagelongine (**5**)^a^		agelongine (**12**) [11]		daminin (**21**) [16]	

	δ_H_, mult. (*J*/Hz)	δ_C_/δ_N_	δ_H_, mult. (*J*/Hz)	δ_C_/δ_N_	δ_H_, mult. (*J*/Hz)	δ_C_/δ_N_

1-N	–	(159)			12.3, s	
2	7.30, d (1.9)	130.2	7.05, d (1.5)	125.6	7.05, d (0.9)	124.6
3	–	94.1	–	94.1	6.16, d (1.9)	109.5
4	6.91, d (1.9)	118.9	6.89, d (1.5)	118.5	6.78, t (1.9; 0.9)	115.7
5	–	121.6	–	123.0	–	120.7
6	–	158.6	–	161.0	–	159.0
8	4.65, t (4.6)	62.6	4.80, t (5.5)	63.6	4.65, t (3.7)	61.8
9	5.07, t (4.6)	59.8	5.07, t (5.5)	61.7	5.01, t (3.7)	58.9
10-N	–	(209)	–		–	
11	9.62, s	146.5	9.42, s	147.4	9.40, s	145.7
12	–	128.0	–	140.2	–	140.9
13	8.95, d (7.9)	145.7	8.99, dt (7.7; 1.5)	147.0	8.78, d (7.4)	144.7
14	8.25, dd (7.9; 6.4)	133.0	8.14, dd (7.7; 6.2)	128.8	8.06, t (7.4; 6.5)	126.6
15	9.26, d (6.4)	146.6	9.05, dt (6.2; 1.5)	146.6	9.04, t (6.5)	144.0
16	–	163.2	–	167.0	–	161.6
17	3.75, s	36.5	–	–	–	–

^a1^H and ^13^C chemical shifts are referenced to the DMSO-*d*_6_ signal (2.50 ppm and 39.5 ppm respectively). ^15^N NMR shifts were not calibrated with an external standard. Therefore, the δ value has an accuracy of about 1 ppm in reference to NH_3_ (0 ppm) and the ^15^N NMR shifts are given without decimals.

The investigation of the ^1^H,^15^N-HMBC spectrum of **5** revealed the correlations H-8, H-11, H-14, and H-15 to N-10 (209 ppm) and suggested the nitrogen N-10 in the aromatic ring which is connected to the aliphatic chain at position C-9 (59.8 ppm). The correlations H-11 and H-15 to C-9 proved this substitution and the correlations H-11 and H-13 to C-16 (163.2 ppm) showed the second substituent to be a formic acid group. The structure of compound **5** was identified to be the *N*-methyl derivative of agelongine (**12**). Debromoagelongine (daminin (**21**) [16]) is the third compound in the agelongine family. All agelongine analogues were isolated from marine sponges.

The citrinamines A–D (**1**–**4**) were further evaluated for antimicrobial and cytotoxic activity. Classical agar diffusion assays were performed using the fungus *Aspergillus niger*, the yeast *Saccharomyces cerevisiae* as well as the Gram-negative bacterium *Escherichia coli*, and the Gram-positive bacterium *Micrococcus luteus* and *Mycobacterium phlei* as test organisms. In agar diffusion assays with *Mycobacterium phlei* considerable inhibition zones were observed for citrinamines B, C, and D (**2**–**4**), while there was also an activity of citrinamine C (**3**) against *Micrococcus luteus*. All compounds (**1**–**4**) showed no inhibition of cell proliferation of mouse fibroblasts.

## Conclusion

The analysis of the marine sponge *Agelas citrina* revealed four new compounds of the pyrrole–imidazole alkaloid (PIA) family. Citrinamines A (**1**) and B (**2**) are closely related to mauritiamine (**7**) which can be seen as the most less complex dimeric PIA (the first published one) in which the monomeric units are only connected by one bond (C-11/C-15’). As it was already described for mauritiamine (**7**) and its congeners nagelamide P (**18**) and nagelamide H (**20**), compounds **1** and **2** were obtained as racemic mixtures. Citrinamines C (**3**) and D (**4**) show a different connection of the two monomeric hymenidin units (C-10/C-15’ and C-15/C-15’) compared to mauritiamine (**7**). Citrinamine C (**3**) is very closely related to nagelamide B (**8**) which are both hydroxylated at C-9 (methoxy in **3**, hydroxy in **8**). The C-15/C-15’ linkage between the imidazole rings of both monomers in citrinamine D (**4**) is uncommon. This connection was only known from the benzosceptrins A–C (**9**–**11**) in which the two imidazole rings are connected by a benzene ring (benzocyclobutane ring system) and not by a single bond as in citrinamine D (**4**). Although, **3** and **4** have different connectivities, both compounds were also obtained as racemic mixtures. Finally, the pyrrole–pyridinium alkaloid *N*-methylagelongine (**5**) was also isolated from *Agelas citrina*. Compound **5** is the *N*-methylated pyrrole derivative of agelongine (**12**). The debromo compound is known as daminin (**21**). The citrinamines A–D (**1**–**4**) were tested against several pathogenic bacteria, fungi, and cultures of mice fibroblasts. Only minor antimicrobial activities were obtained for citrinamines B–D (**2**–**4**) whereas no activities were found in the cytotoxicity assay for citrinamines A–D (**1**–**4**).

## Experimental

### General experimental procedures

^1^H, ^13^C, and ^15^N NMR spectra were conducted on Bruker Avance I 400 MHz, Bruker Avance II 600 MHz, and Bruker Avance III 850 MHz NMR spectrometers. All experiments were measured at 303 K in DMSO-*d*_6_ as solvent. The DQF-^1^H,^1^H-COSY, ^1^H,^13^C-HSQC, ^1^H,^13^C-HMBC, ^1^H,^15^N-HSQC, ^1^H,^15^N-HMBC, and ^1^H,^1^H-NOESY experiments were carried out using standard parameters. The mixing time for NOESY spectra was set to 200 ms, and the delay for the HMBC measurements was set to 80 ms. HPLC-MS analysis were performed with an Agilent 1100 HPLC systems and Bruker Daltonics micrOTOF_LC_. Analytical chromatography: Waters XTerra RP_18_ column (3.0 mm × 150 mm, 3.5 µm) with a MeCN/H_2_O/HCOOH gradient [0 min: 10% MeCN/90% HCOOH (0.1%); 30 min: 60% MeCN/40% HCOOH (0.1%) with a flow rate of 0.4 mL min^−1^]. Preparative chromatography: Prontosil Eurobond C_18_ column (20 mm × 250 mm, 5 µm) with a MeCN/TFA (0.1%) gradient. UV spectra were recorded during HPLC analysis with a DAD (Agilent).

#### Animal material

The marine sponge *Agelas citrina* was collected by SCUBA diving on March 11, 2001 at San Salvador in the Bahamas (27 m depth). The samples were immediately frozen after collection and kept at −20 °C until extraction. A voucher specimen was deposited under registration no. ZMA POR. 17278 at the Zoölogical Museum, University of Amsterdam (The Netherlands). Sponge identification was kindly conducted by W. H. de Weerdt and Dr. R. W. M. van Soest, Institute for Biodiversity and Ecosystem Dynamics, Zoölogical Museum, University of Amsterdam, The Netherlands (new address of RWMvS: Netherlands Centre for Biodiversity, Department Marine Zoology, Leiden, The Netherlands).

#### Extraction and isolation

As already described in [17] the freeze-dried sponge tissue of *Agelas citrina* (120 g) was crushed with a mill and extracted exhaustively at room temperature with a 1:1 mixture of MeOH/CH_2_Cl_2_. Part of the crude extract (40.84 g) was partitioned between *n*-hexane (4 × 600 mL) and MeOH (450 mL). After evaporating, the MeOH extract was then partitioned between *n*-BuOH (3 × 600 mL) and H_2_O (450 mL). The resulting *n*-BuOH phase (22.02 g) from the solvent partitioning scheme was purified by gel chromatography on Sephadex LH-20 (Pharmacia) using MeOH as mobile phase. The final purification of the isolated compounds was achieved by preparative RP_18_ HPLC on a Prontosil Eurobond C_18_ column (20 mm × 250 mm, 5 µm) applying a MeCN/TFA (0.1%) gradient to yield **1** (9.2 mg, 0.0077% of dry weight), **2** (4.0 mg, 0.0033%), **3** (1.7 mg, 0.0014%), **4** (5.1 mg, 0.0043%), **5** (1.3 mg, 0.0011%), **6** (112.8 mg, 0.0940%), **14** (56.3 mg, 0.0469%), and **15** (42.7 mg, 0.0356%). For compounds **1**–**4** a second preparative chromatography was carried out.

**Citrinamine A (1):** light-yellow powder; UV (DAD): λ_max_ = 228, 270 nm; no CD effect (MeOH) was obtained (λ 210 to 300 nm); ^1^H and ^13^C NMR data in Table 1; HPLC/HR(+)ESIMS: *t*_R_ = 16.4 min, *m*/*z* = 633.0330 [M + H]^+^ (calcd. for C_22_H_23_Br_2_N_10_O_3,_ 633.0316), Δ*m* = 2.9 ppm.

**Citrinamine B (2):** light-yellow powder; UV (DAD): λ_max_ = 270 nm; no CD effect (MeOH) was obtained (λ 210 to 300 nm); ^1^H and ^13^C NMR data in Table 2; HPLC/HR(+)ESIMS: *t*_R_ = 15.4 min, *m*/*z* = 740.0337 [M + H]^+^ (calcd. for C_24_H_28_Br_2_N_11_O_5_S, 740.0357), Δ*m* = 2.7 ppm.

**Citrinamine C (3):** light-yellow powder; UV (DAD): λ_max_ = 239, 272 nm; no CD effect (MeOH) was obtained (λ 210 to 300 nm); ^1^H and ^13^C NMR data in Supporting Information File 1, Table S1; HPLC/HR(+)ESIMS: *t*_R_ = 17.7 min, *m*/*z* = 650.0493 [M + H]^+^ (calcd. for C_23_H_26_Br_2_N_9_O_4_, 650.0469), Δ*m* = 2.9 ppm.

**Citrinamine D (4):** light-yellow powder; UV (DAD): λ_max_ = 242, 269 nm; no CD effect (MeOH) was obtained (λ 210 to 300 nm); ^1^H and ^13^C NMR data in Supporting Information File 1, Table S1; HPLC/HR(+)ESIMS: *t*_R_ = 16.7 min, *m*/*z* = 650.0470 [M + H]^+^ (calcd. for C_23_H_26_Br_2_N_9_O_4_, 650.0469), Δ*m* = 0.2 ppm.

***N*-Methylagelongine (5):** light-yellow powder; UV (DAD): λ_max_ = 242, 274 nm; ^1^H and ^13^C NMR data in Table 3; HPLC/HR(+)ESIMS: *t*_R_ = 11.9 min, *m*/*z* = 353.0135 [M + H]^+^ (calcd. for C_14_H_14_BrN_2_O_4_, 353.0131), Δ*m* = 1.1 ppm.

#### Antimicrobial assay

As already described in [18] the antimicrobial activities were determined by agar diffusion tests using paper disks of 6 mm diameter soaked with 20 µL of the test compound in MeOH (1 mg/mL). The microorganisms were obtained from the HZI collection, grown on standard media, and cultured in liquid agar medium to a final OD of 0.01 (bacteria) or 0.1 (yeasts). Spores of fungi were collected from well-grown Petri dishes, which were rinsed with 10 mL of sterile H_2_O. One mL of the spore suspension was added to 100 mL of molten agar medium. Plates were incubated at 30 °C, and the diameters of resulting inhibition zones were measured after 1 and 2 days.

#### Cell proliferation assay

As already described in [18] L929 mouse fibroblasts were obtained from the Deutsche Sammlung von Mikroorganismen und Zellkulturen (DSMZ) and cultivated at 37 °C and 10% CO_2_ in DME medium (high glucose) supplemented with 10% fetal calf serum. Cell culture reagents were purchased from Life Technologies Inc. (GIBCO BRL). Growth inhibition was measured in microtiter plates. Aliquots of 120 µL of the suspended cells (50000/mL) were added to 60 µL of serial dilutions of the test compounds. After 5 days the growth was determined using an MTT assay [19].

## Supporting Information

File 1 NMR data.
