# Supplementary material for: The marine sponge Agelas citrina as a source of the new pyrrole–imidazole alkaloids citrinamines A–D and N-methylagelongine
Source: Beilstein J Org Chem. 2015 Oct 29;11:2029–37. doi: 10.3762/bjoc.11.220 (PMC4660978; doi:10.3762/bjoc.11.220)
Supplement: File 1 — NMR data. [file Beilstein_J_Org_Chem-11-2029-s001.pdf]

Supporting Information

for

The marine sponge *Agelas citrina* as a source of the new pyrrole–imidazole alkaloids citrinamines A–D and *N*-methylagelongine

Christine Cychon, Ellen Lichte and Matthias Köck*

Address: Alfred-Wegener-Institut, Helmholtz-Zentrum für Polar- und Meeresforschung, Am Handelshafen 12, 27570 Bremerhaven, Germany

Email: Matthias Köck - mkoeck@awi.de

*Corresponding author

NMR data

Figure S1. 1D ^{1}H-NMR spectrum of citrinamine A (**1**) in DMSO-d_6 .... S2

Figure S2. 1D ^{13}C-NMR spectrum of citrinamine A (**1**) in DMSO-d_6 .... S2

Figure S3. 1D ^{1}H-NMR spectrum of citrinamine B (**2**) in DMSO-d_6 .... S3

Figure S4. 1D ^{13}C-NMR spectrum of citrinamine B (**2**) in DMSO-d_6 .... S3

Figure S5. ^{1}H,^{13}C-HMBC and the structure of citrinamine C (**3**) S4

Table S1. ^{1}H, ^{13}C, and ^{15}N chemical shifts of citrinamines C (**3**) and D (**4**) S5

Figure S6. 1D ^{1}H-NMR spectrum of *N*-methylagelongine (**5**) in DMSO-d_6 .... S6

Figure S7. 1D ^{13}C-NMR spectrum of *N*-methylagelongine (**5**) in DMSO-d_6 .... S6

Citrinamine A (1)

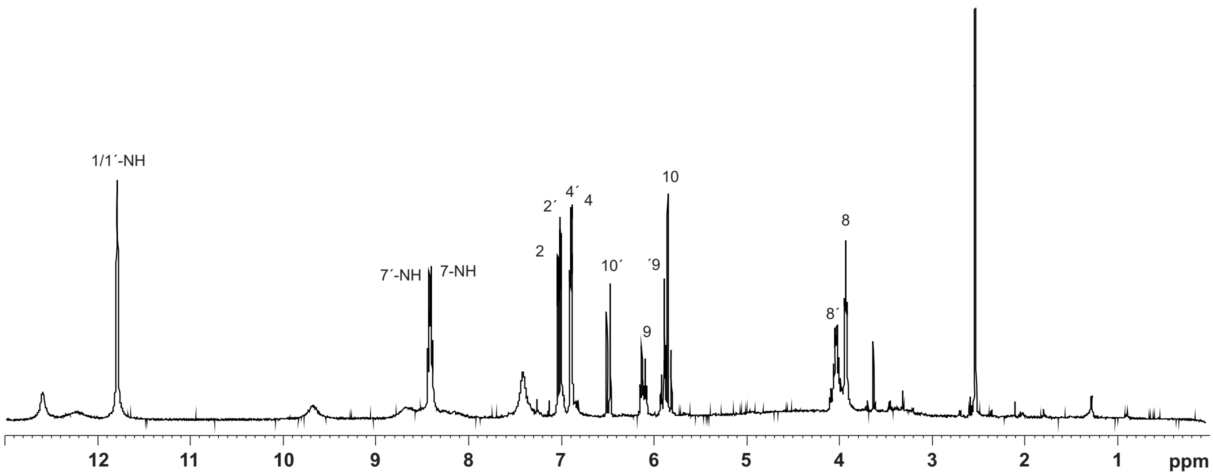

Figure S1. 1D ^{1}H NMR spectrum of citrinamine A (**1**) in DMSO-d_6, 303 K, 400 MHz.

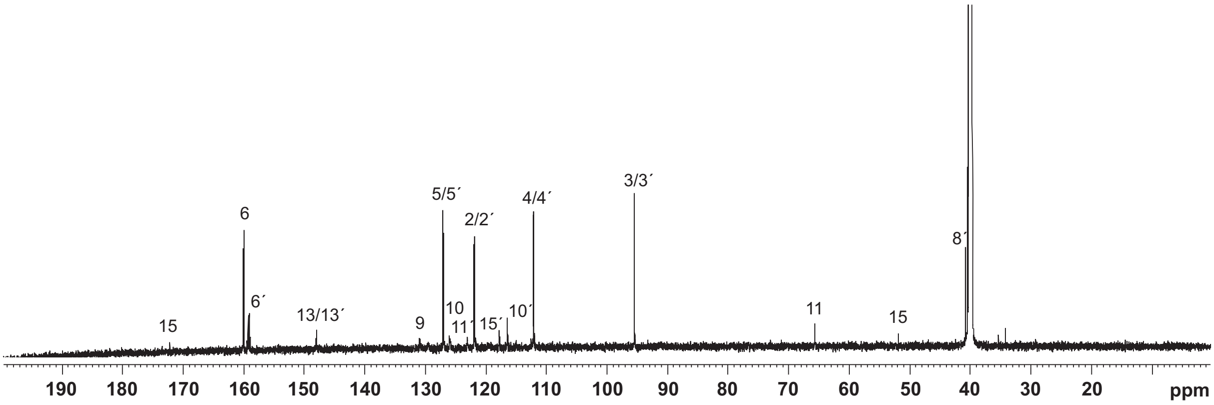

Figure S2. 1D ^{13}C-NMR spectrum of citrinamine A (**1**) in DMSO-d_6, 303 K, 850 MHz.

Citrinamine B (2)

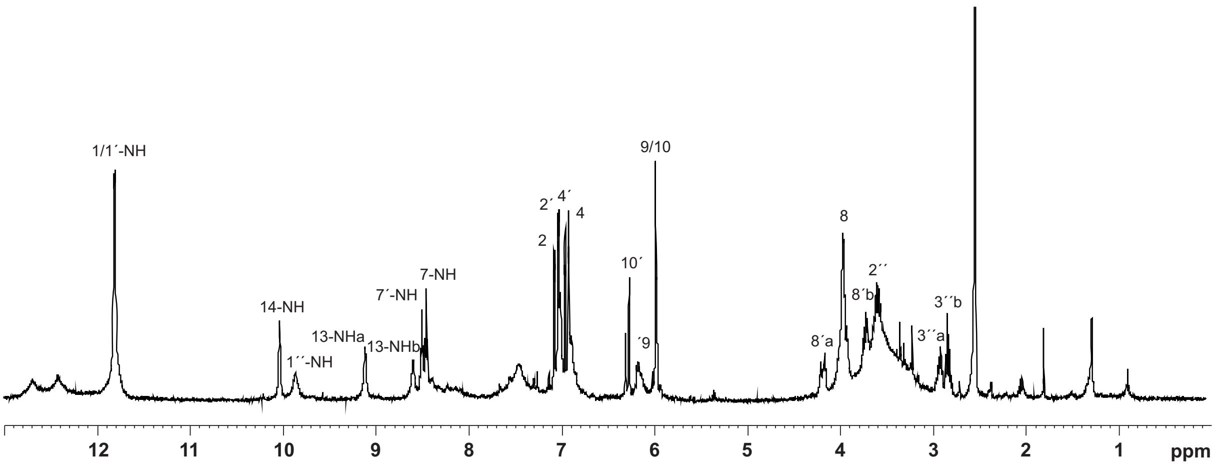

Figure S3. 1D ^{1}H-NMR spectrum of citrinamine B (**2**) in DMSO-d_6, 303 K, 400 MHz.

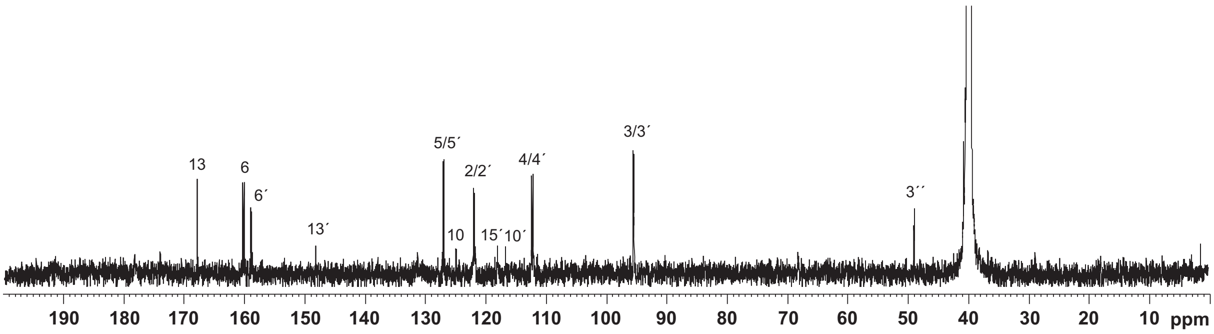

Figure S4. 1D ^{13}C-NMR spectrum of citrinamine B (**2**) in DMSO-d_6, 303 K, 850 MHz.

Citrinamines C (3) and D (4)

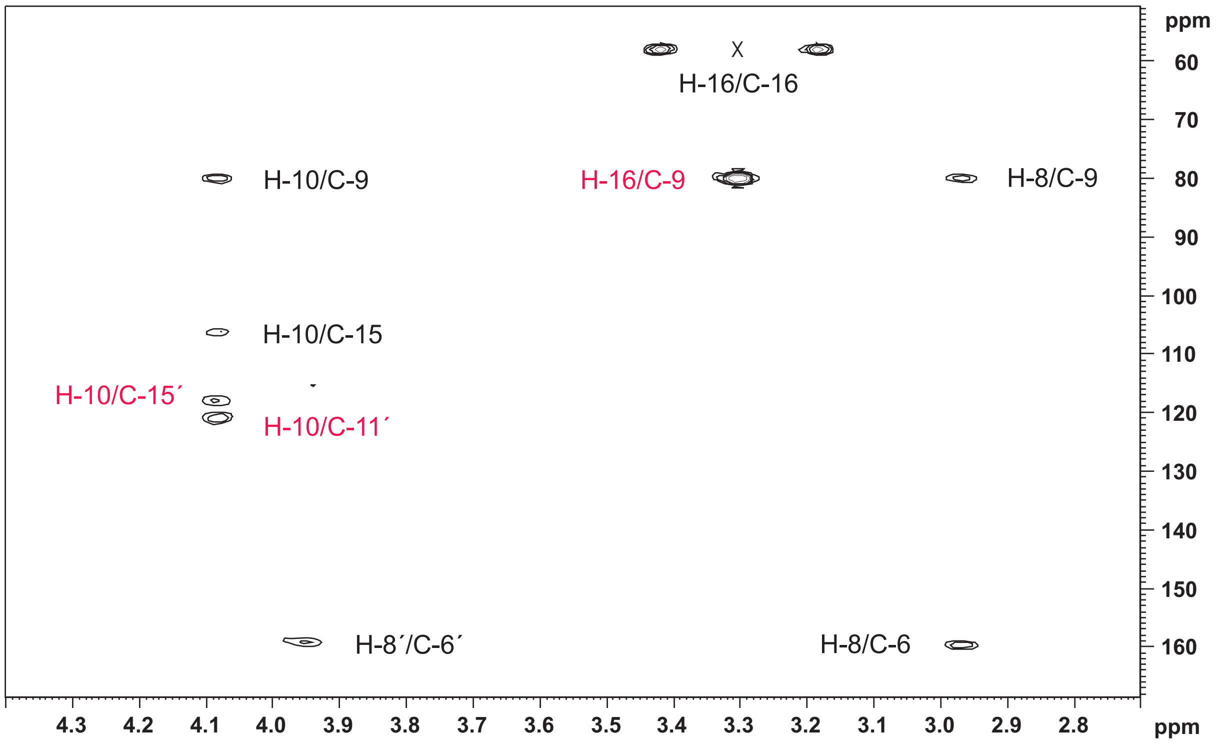

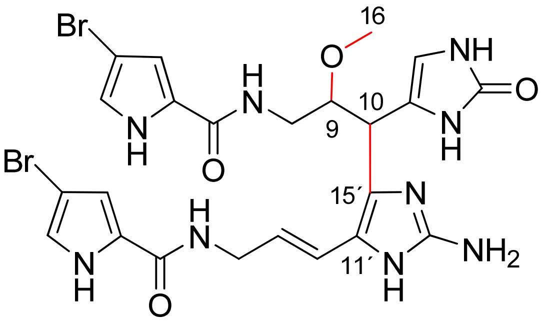

3

Figure S5. $^{1}H,^{13}C$-HMBC and the structure of citrinamine C (**3**) (key correlations and bonds in red).

Table S1. ^{1}H, ^{13}C, and ^{15}N chemical shifts of citrinamines C (**3**) and D (**4**) (600 MHz, DMSO-d_6).[a]

Position	citrinamine C (**3**)		citrinamine D (**4**)	
	δ_H, mult. (J/Hz)	δ_C / δ_N	δ_H, mult. (J/Hz)	δ_C / δ_N
1-NH	11.81, s	(161)	11.78, s	(161)
2	6.98, m	121.2	6.98[b]	121.2
3	-	94.9	-	94.8
4	6.86, m	111.1	6.88[b]	111.7
5	-	126.6	-	126.5
6	-	159.9	-	159.3
7-NH	8.22, t (5.8)	(102)	8.06, t (5.9)	(103)
8	3.45[b]; 2.96, m	42.2	3.26[b] ; 3.20[b]	41.5
9	3.72, m	80.8	3.45, m	78.1
10	4.09, d (4.8)	34.5	2.34, d (6.3)	27.2
11	-	117.6	-	120.5
12-NH	9.66, s	(127)	10.20, d (2.3); 10.00, d (2.3)	(137 / 130)
13	-	154.6	-	153.6
14-NH	9.74, s	(131)	10.20, d (2.3); 10.00, d (2.3)	(137 / 130)
15	6.38, s	106.4	-	105.5
16	3.31, s	58.5	3.18, s	56.8
1´-NH	11.81, s	(161)	11.81, s	(161)
2´	6.98, m	121.2	6.97[b]	121.0
3´	-	94.9	-	94.8
4´	6.86, m	111.1	6.87[b]	111.5
5´	-	126.6	-	126.5
6´	-	159.3	-	159.6
7´-NH	8.37, t (5.7)	(106)	8.37, t (5.7)	(106)
8´	3.93, m	40.8	3.95, dd (5.3)	40.0
9´	6.05, m	127.4	6.16[b]	127.7
10´	6.35, d (16.0)	116.6	6.14[b]	116.3
11´	-	121.3	-	121.5
12´-NH	12.48, s	(130)		
13´	-	147.1	-	154.2
13´-NH_2				
14´-NH	11.79, s	(148)		
15´	-	117.9	-	115.8

[a] ^{1}H and ^{13}C chemical shifts are referenced to the DMSO-d_6 signal (2.50 ppm and 39.5 ppm, respectively). ^{15}N NMR shifts were not calibrated with an external standard. Therefore, the δ value has an accuracy of about 1 ppm in reference to NH_3 (0 ppm) and the ^{15}N NMR shifts are given without decimals.

[b] No multiplicity information could be given because of overlapped signals.

N-Methylagelongine (5)

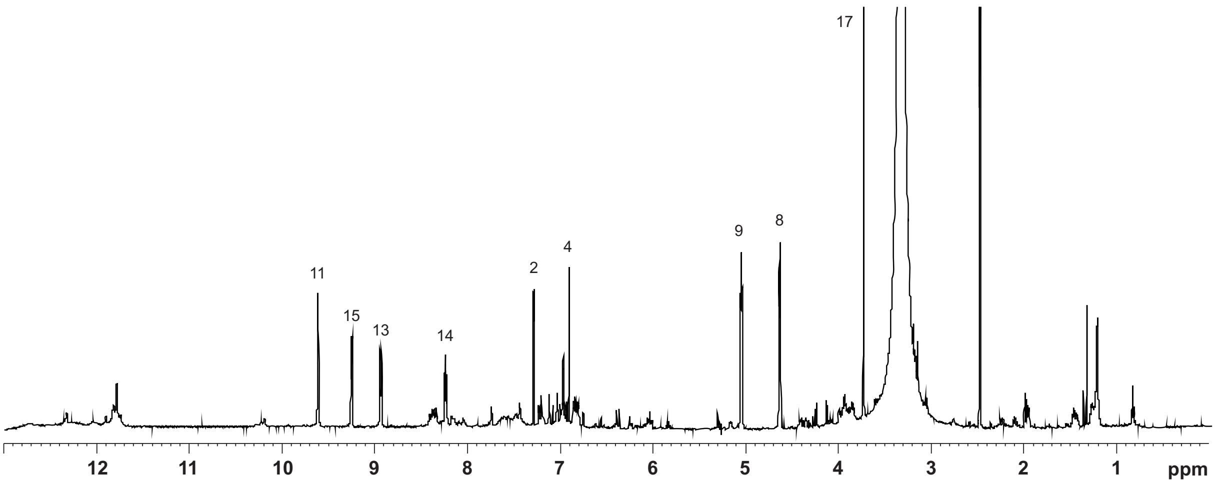

Figure S6. 1D ^{1}H-NMR spectrum of *N*-methylagelongine (**5**) in DMSO-d_6, 303 K, 600 MHz.

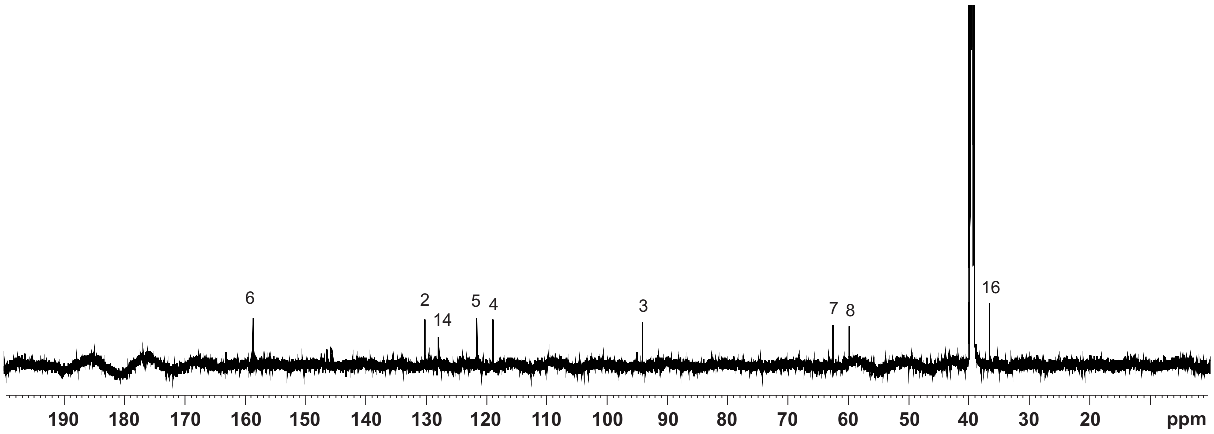

Figure S7. 1D ^{13}C-NMR spectrum of *N*-methylagelongine (**5**) in DMSO-d_6, 303 K, 600 MHz.
